# Surface Engineering of MXene and Functional Fullerenols for Cancer Biomarker ‘eIF_3_d’

**DOI:** 10.1021/acs.langmuir.5c00157

**Published:** 2025-03-21

**Authors:** Dilek Soyler, Volkan Dolgun, Oyku Cetin, Yaqoob Khan, Emine Guler Celik, Salih Ozcubukcu, Husnu Emrah Unalan, Suna Timur, Saniye Soylemez

**Affiliations:** † Department of Biomedical Engineering, Faculty of Engineering, Necmettin Erbakan University, Konya 42090, Türkiye; ‡ Department of Chemistry, Faculty of Science, Middle East Technical University, Ankara 06800, Türkiye; § Department of Metallurgical and Materials Engineering, Faculty of Engineering, Middle East Technical University, Ankara 06800, Türkiye; ∥ Department of Bioengineering, Faculty of Engineering, Ege University, Bornova, Izmir 35100, Türkiye; ⊥ Department of Biochemistry, Faculty of Science, Ege University, Bornova, Izmir 35100, Türkiye; # Central Research Testing and Analysis Laboratory Research and Application Center, Ege University, Bornova, Izmir 35100, Türkiye

## Abstract

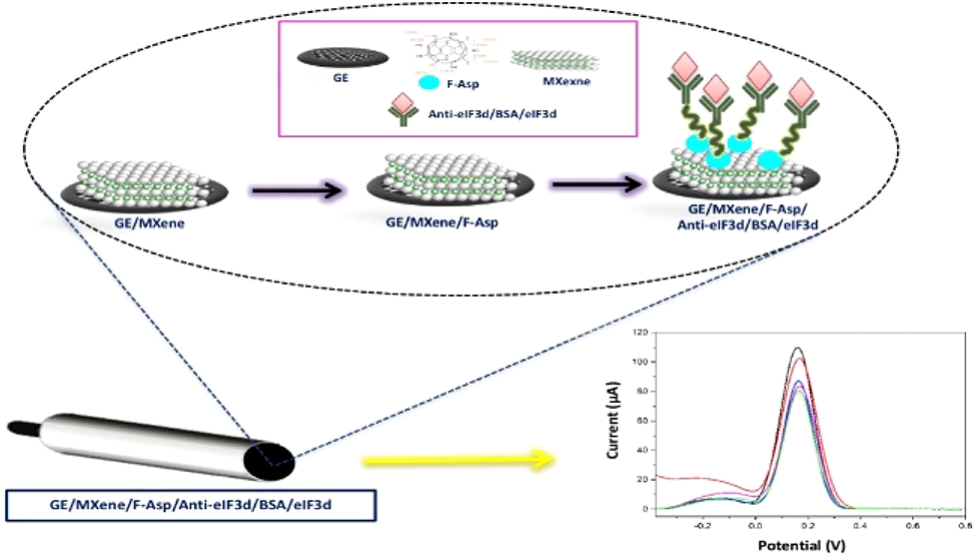

Selective and sensitive detection of eIF_3_d (eukaryotic translation initiation factor 3 complex, subunit D), a protein biomarker, is of fundamental significance for the diagnosis of various cancers. Here, we report an electrochemical sensor based on MXene and aspartic acid-functionalized fullerenol (F-Asp) for the biosensing of eIF_3_d. To construct such an innovative sensing platform, MXene was first synthesized, followed by the convenient functionalization of fullerenol with aspartic acid groups (F-Asp) through hydroxylation and activation of fullerenes. Finally, a bioplatform was created for eIF_3_d sensing by modifying the graphite electrode (GE) surface with MXene and F-Asp, followed by surface functionalization with anti-eIF_3_d antibody via EDC/NHS chemistry. Detailed electrochemical and analytical material characterization methods were utilized after each surface modification step. Notably, the surface-engineered MXene:F-Asp showed superior electrochemical features. The sensor’s response to eIF_3_d was achieved in the linear range of 10 to 250 ng/mL, with a detection limit of 0.14 ng/mL. The selectivity of the sensor was assessed by monitoring its response to eIF_3_d in the presence of a variety of interfering compounds. Analysis of eIF_3_d was effectively performed in synthetic serum samples. The promising electrochemical sensing properties of the designed sensor suggest great potential for various real-time health monitoring applications.

## Introduction

In clinical trials, interactions between antigens and antibodies are commonly utilized to diagnose, treat, and predict the prognosis of different types of cancer. A subset of mRNAs has a 7-methylguanosine cap that eIF_3_d can particularly recognize and bind to; these interactions are thought to be crucial for controlling cell proliferation. It has been found that down-regulating eIF_3_d is linked to preventing the growth of cancer cells, suggesting that it has predictive value in cancer diagnosis.^1,2^ The identification of the eIF_3_d protein, a recently identified possible clinical biomarker for a number of cancer types, has enormous potential for use in cancer diagnostics. According to recent research, eIF_3_d is overexpressed in a number of cancerous tumors, such as melanoma,^3^ prostate cancer,^4^ and colon cancer.^5^ In a number of cancer types, eIF_3_d has also been identified as a possible therapeutic target.^2,3,6^ Recent data suggest that dysregulated eIF_3_d expression plays a significant role in cancer and various genetic disorders, highlighting the urgent need for innovative detection strategies. This underscores the growing demand for advances in cancer biomarker screening and detection.

As of today, a number of analysis platforms have been documented for the quick identification of different biomarkers.^7−13^ These include fluorescence,^7^ chromogenic,^8^ other biochemical-based analysis like enzyme-linked immunosorbent assay (ELISA),^9^ surface acoustic wave (SAW),^10^ electrochemical (EC),^11^ surface-enhanced Raman scattering (SERS),^12^ and colorimetric analysis.^13^ Unfortunately, some of these techniques involve complex multistep manufacturing processes, limiting their applicability due to the need for specialized expertise and expensive equipment. Consequently, the monitoring and screening of eIF_3_d in bodily fluids have garnered significant attention. In this field, electrochemically based biosensing strategies are fast and straightforward. It is well-acknowledged that the limitations of traditional detection methods can be addressed by these innovative electrochemical nanobiosensors. For a variety of analytes, including proteins, nucleic acids, small compounds, and even cells, electrochemical biosensors with biological recognition components and electronic transducers can serve as reliable analytical instruments.^14^ Electrochemical biosensors convert biorecognition events into quantifiable signals, which are analyzed using appropriate electrochemical techniques based on the electroactive nature of the analytes.^15^ Because of their significant efficiency, quick reaction, remarkable sensitivity, and specificity, electrochemical biosensors are becoming more and more recognized as effective instruments for cancer diagnosis, especially in liquid biopsies. In addition, their ease of portability and miniaturization makes them applicable for point-of-care testing to acquire physiological and biochemical information. Analysis of cancer biomarkers, including tumor-associated nucleic acids, tumor protein markers, extracellular vesicles (EVs), and tumor cells, is of particular interest among the many applications of electrochemical biosensors. This is because it highlights the potential of these biosensors to detect the presence of cancer and track the progress of the disease.

Carbon-based materials are a prominent focus in biomedical and sensor applications and are valued for their excellent conductivity and unique optical properties. Among different carbon-based nanomaterials, fullerenols (FLs) (fullerene derivatives) have attracted significant interest due to their high solubility in water (and polar solvents) and stability. They possess notable photo and electron-acceptor features, making them essential for organic electronic applications, including high conductivity, electron mobility, and fluorescence quenching properties.^16−18^ Previously, we showed that amino acid-functionalized fullerenol derivatives were identified as a novel artificial enzyme model^19^ and were employed as enzyme substitutes to promote osteogenic differentiation in stem cells^20^ and sensor systems for acetylcholine detection.^21^ Due to their superior optical and electrical characteristics and appropriate structural morphologies, F-Asp-based nanobiosensors are being developed as easy-to-use tools and replacements for highly sensitive biosensors. The presence of amino acid groups in the fullerenol structure is advantageous, as they enable easy modification of the electrode surface with the target anticor group through covalent bonding.

Two-dimensional titanium carbide (Ti_3_C_2_T_*x*_) MXene has emerged as a promising nanomaterial for cancer biomarker detection due to its remarkable electrical conductivity, large surface area, and ease of biomolecule immobilization.^22,23^ The emergence of MXene-based materials has led to an increase in studies in the field of electronics due to their excellent electrochemical performance, large surface, outstanding mechanical properties, and unique interfacial chemistry, where their performance can be enhanced through multi-interface engineering.^24,25^ In particular, Ti_3_C_2_T_*x*_ MXene offers high sensitivity and selectivity for biosensor applications. Studies have shown that MXene-based materials, such as reduced graphene oxide–MXene–multiwalled carbon nanotube electrodes and Ti_3_C_2_T_*x*_ MXene with manganese oxide, are highly effective and selective for detecting hydrogen peroxide, a key biomarker released by cancer cells.^26,27^ Additionally, “turn-on” nanobiosensors based on MXene and DNA-silver nanoclusters have achieved ultrasensitive detection of miRNA-191, a key cancer biomarker.^28^ Moreover, hybrid structures combining MXene with gold nanoparticles have provided high stability and low detection limits for electrochemical biomarker detection^29^ highlighting their transformative potential in early cancer diagnostics.

**Scheme 1:**
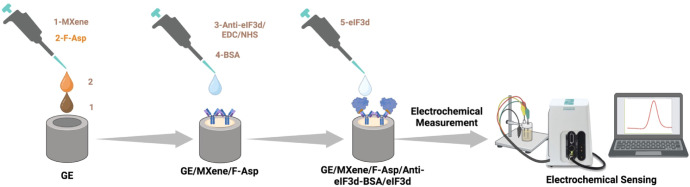
Schematic Illustration of the Fabrication Process for an Electrochemical Nanobiosensor Based on MXene-F-Asp Nanostructures for eIF_3_d Sensing

Herein, interfacial chemistry takes place within MXene, its composite with fullerenol (F-Asp), and the eIF_3_d protein, which are deposited one on top of another onto an electrode surface for antibody/receptor interactions. For this purpose, the eIF_3_d protein is chosen as a potential biomarker for cancer diagnosis. To the best of our knowledge, the combination of MXene and F-Asp, as well as the detection of the eIF_3_d biomarker using MXene-F-Asp-based electrochemical nanosensor technology, has not been previously reported. Consequently, this work is a strong candidate for the application of MXene-F-Asp-modified electrodes as an immunosensor platform that offers a surface capable of effectively immobilizing biomolecules. The developed sensing procedure is simple, cost-effective, and rapid, requiring neither specialized expertise nor complex equipment. Scheme 1 shows the process of nanobiosensor fabrication utilized in this work.

## Material and Methods

### Reagents and Apparatus

Graphite electrodes, *N*-(3-(dimethylamino)propyl)-*N*′-ethylcarbodiimide hydrochloride (EDC), *N*-hydroxysuccinimide (NHS), eIF_3_d antibody (Prestige Antibodies), eIF_3_d, alpha-fetoprotein (AFP), human epidermal growth factor receptor 2 (HER2) antigen (PrEST Antigens), bovine serum albumin (BSA) (lyophilized powder, ≥96% (agarose gel electrophoresis)), disodium hydrogen phosphate, sodium phosphate monobasic, and potassium chloride were purchased from Sigma Chemical Company (St. Louis, MO, USA).

The AUTOLAB PGSTAT 204 Analysis System (NOVA software, Metrohm, The Netherlands) was utilized to perform electrochemical measurements, such as differential pulse voltammetry (DPV), cyclic voltammetry (CV), and electrochemical impedance spectroscopy (EIS). Electrochemical experiments were conducted using graphite as the working electrode, Ag/AgCl (3.0 M KCl) as the reference electrode, and platinum (Pt) as the counter electrode. Electrochemical investigations were conducted in a 10 mL reaction cell under ambient conditions with 5.0 mM Fe(CN)_6_^3–/4–^ as a redox probe. DPV and CV measurements were performed in a potential range of −0.4 to 0.8 V. EIS measurements were performed within the frequency range of 0.02–10 Hz at 0.18 V.

The morphological analysis of the fabricated Ti_3_C_2_T_*x*_ MXene was conducted using a field-emission scanning electron microscope (SEM, Nova NanoSEM 430) at an operating voltage of 20 kV and high-resolution transmission electron microscopy (HR-TEM, JEOL JEM-2100F) operated at 200 kV. For further analysis, X-ray photoelectron spectroscopy (XPS) measurements were carried out using a SPECS PHOIBOS instrument, with the C 1s peak at 284.8 eV used as the reference.

For biosensing experiments, survey and high-resolution scans of samples were analyzed by X-ray photoelectron spectroscopy (XPS; Thermo Scientific, Al–Kα hv = 1486.6 eV, equipped with a multichannel detector). Survey spectra (with a pass energy of 200 eV and a step size of 1 eV) and high-resolution scans (with a pass energy of 50 eV and a step size of 0.05 eV) were acquired for each analysis. The K-Alpha instrument uses internal standard samples, such as copper, silver, and gold, to automatically calibrate the XPS binding energy scale. Calibration was carried out using the Au 4f7/2 (84.1 eV), Cu 2p3/2 (932.2 eV), and Ag 3d5/2 (368.2 eV) reference lines. The surface morphology of the fabricated biosensors was examined using field emission scanning electron microscopy (FE-SEM) (ZEISS GeminiSEM 500, Jena, Germany).

### Fabrication of Ti_3_AlC_2_ MAX Phase

The synthesis of the Ti_3_AlC_2_ MAX phase followed the methods reported in our previous studies.^30,31^ Briefly, titanium (Ti), aluminum (Al), and graphite (C) powders served as the raw materials (Ti and Al from Nanografi, Türkiye; C from Fisher Scientific, USA). The powder mixture, with a stoichiometric ratio of 3:1:2, was prepared by ball milling. The resultant powder mixture was sintered in a tubular furnace under a continuous argon gas flow. The temperature of the furnace was increased at a rate of 5 °C/min until reaching 1500 °C, where it was maintained for 3 h and then allowed to cool gradually to room temperature. The sintered product, Ti_3_AlC_2_ MAX, was then crushed into powders to ease further processing.

### Production of Ti_3_C_2_T_*x*_ MXene Nanosheets

A liquid chemical etching process enabled the selective removal of the Al layer from the Ti_3_AlC_2_ MAX phase to obtain Ti_3_C_2_T_*x*_ MXene nanosheets. For the etching process, 3.2 g of lithium fluoride (LiF) powder (Sigma-Aldrich, USA) was dissolved in 40 mL of 9 M hydrochloric acid (HCl) (Sigma-Aldrich, USA) in a high-density polyethylene (HDPE) container. Then, 2 g of the Ti_3_AlC_2_ MAX powder was gradually added to the LiF-acid solution. The dispersion was stirred at 3500 rpm and kept at 35 °C for 24 h. After 24 h, the mixture was washed with deionized (DI) water until the pH became neutral. The delaminated MXene nanosheets were separated from the unreacted MAX phase by centrifugation and collected as the supernatant.

### Synthesis of Fullerenol

**Scheme 2:**
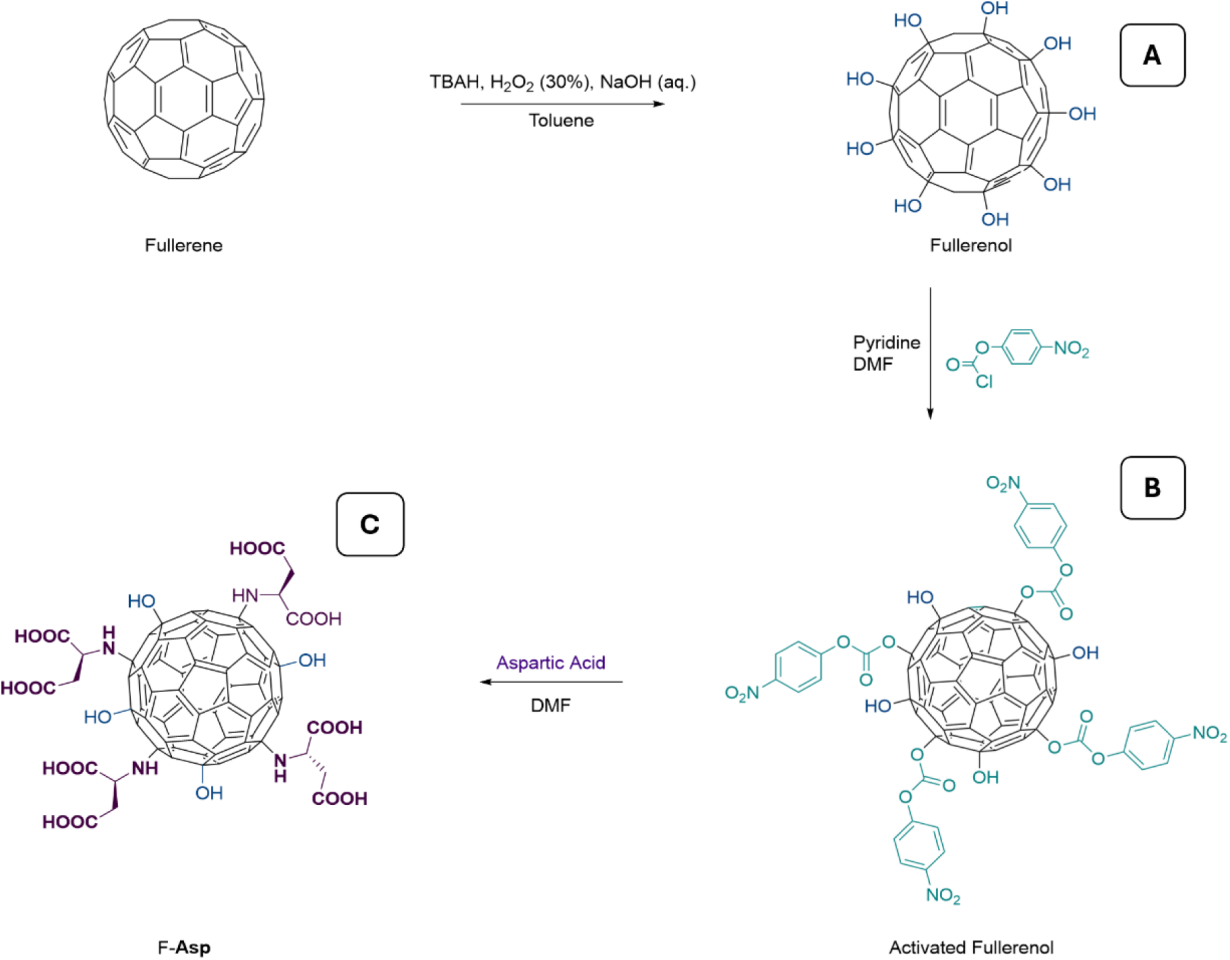
Synthetic Pathway of (A) Fullerenol, (B) Activated Fullerenol, and (C) F-Asp

To a solution of fullerene-C_60_ (160 mg, 0.2 mmol) in toluene (100 mL), NaOH solution (4 mL, 1 g/mL) was added. Twelve drops of 30% H_2_O_2_ solution and, as a phase transfer catalyst, 0.5 mL of tetrabutylammonium hydroxide (TBAH) were added. The final solution was stirred for 5 days at room temperature. After completion of the reaction, the solvent was decanted, and 50 mL of ethanol was added to the dark brown solid. The suspension was further stirred at room temperature for 30 min to remove excess TBAH. The product was precipitated and freeze-dried. A dark brown solid product (200 mg) was obtained with a yield of 86%. The resulting product was characterized by its increasing solubility in water (Scheme 2A).

### Synthesis of Activated Fullerenol

A 120 mg amount of fullerenol was suspended in DMF and sonicated in an ultrasonic bath for an hour. The suspension was cooled to 0 °C, and *p*-nitrophenylchloroformate (800 mg), anhydrous pyridine (4 mL), and catalytic *N*,*N*-dimethylaminopyridine (DMAP, 40 mg) were added. The final solution was stirred for 2 days under a nitrogen atmosphere, with an hour of sonication in the ultrasonic bath performed twice a day. After completion of the reaction, the obtained black solid was precipitated by the addition of diethyl ether. The solid was washed with diethyl ether and dichloromethane (DCM) and isopropyl alcohol via centrifugation (4500 rpm, 5 min) to remove impurities. The resulting product was characterized by its increasing solubility in DMF (Scheme 2B).

### Synthesis of Fullerenol Aspartic Acid (F-Asp)

**Figure 1:**
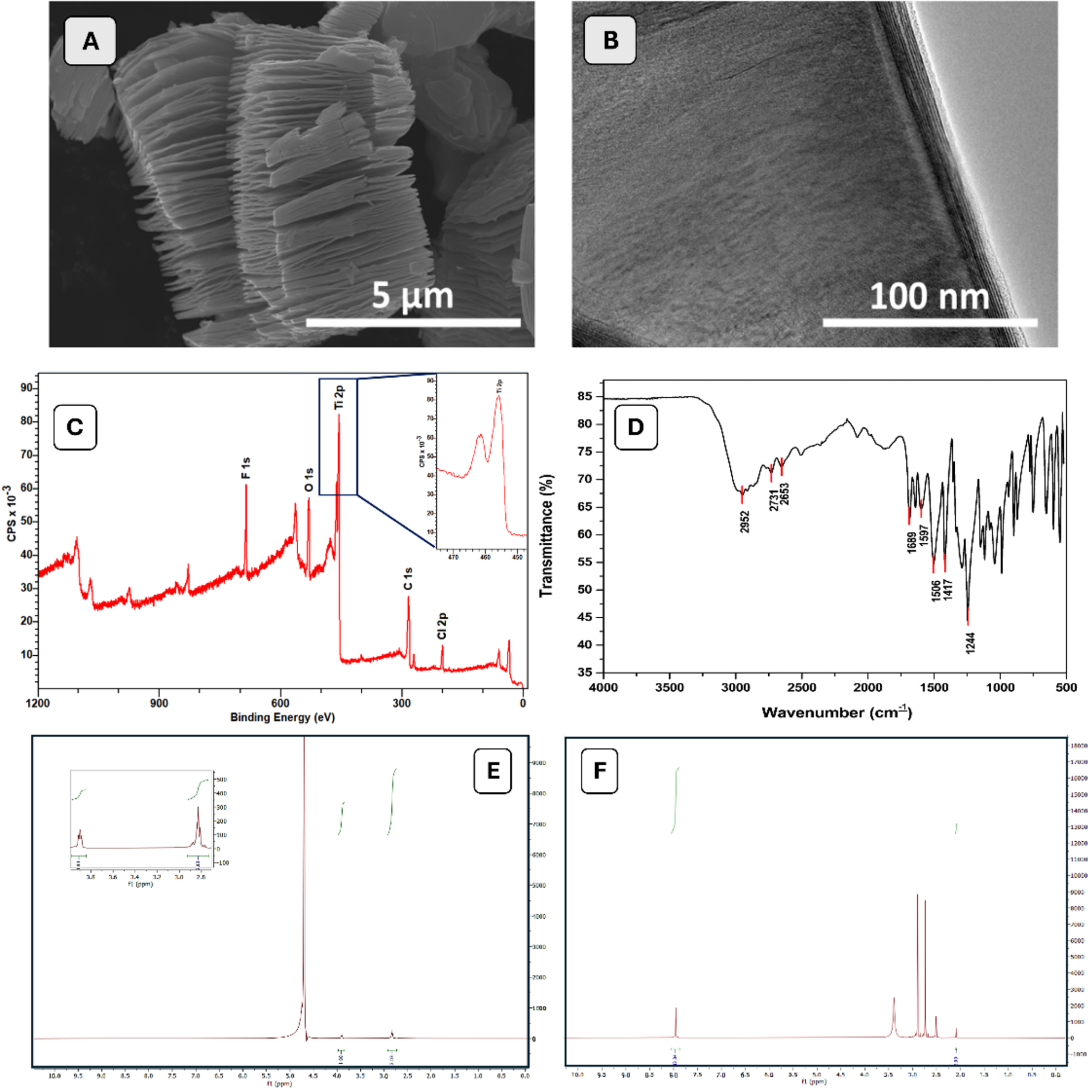
(A) SEM image of as-prepared Ti_3_C_2_T_*x*_ MXene before delamination, showing the accordion-like structure. (B) HR-TEM image highlighting the well-defined layered structure with distinct interlayer spacing. (C) XPS spectra of freshly prepared Ti_3_C_2_T_*x*_ MXene with the labels of Ti 2p, C 1s, O 1s, F 1s, and Cl 2p labels (inset: zoom-in to the Ti 2p peak). (D) IR spectrum of F-Asp (E) ^1^H NMR Spectrum of F-Asp. ^1^H NMR (400 MHz, D_2_O) δ 3.89 (t, *J* = 6.7 Hz. 1H(α-CH)), 2.87–2.79 (m, 2H(β-CH_2_)), and (F) ^1^H NMR Spectrum of F-Asp including DMF as an internal standard in DMSO-*d*_*6*_.

l-Aspartic acid (40 mg) was added to a suspension of 30 mg of activated fullerenol in DMF that had been sonicated for 45 min. The black solution was stirred under nitrogen for 2 days at room temperature, with 45 min of sonication twice a day. The brown solid was precipitated out with cold diethyl ether following the completion of the reaction. The obtained solid was washed four times with methanol: DCM solution (50:50) by centrifuging for 5 min at 4500 rpm. The brown precipitate was then dried, and 24 mg of F-Asp was obtained (Scheme 2C). It was characterized by using ^1^H NMR and IR spectroscopy (Figure 1D–F).

### Electrode Fabrication and Surface Functionalization

After the bare GE cleaning procedure with emery paper, GE was stabilized by scanning the potential between −1.8 V and +1.8 V via cyclic voltammetry, with a scan rate of 100 mV/s for 20 cycles in PBS (pH 7.4). The GE/MXene/F-Asp was produced as follows: 3.0 μL of 1/2 diluted MXene solution was dropped onto the GE surface, and the electrode was allowed to dry at room temperature for 1 h. Then, 10.0 μL of F-Asp (1.0 mg/mL) was dropped onto the modified surface, and the electrode was left to dry under ambient conditions for 1 h. For 90 min, the electrode was immersed in a solution of EDC (50 mM) and NHS (50 mM) at pH 6.0 in PBS. Following a PBS (pH 6.0) wash of the activated surfaces, 10 μg/mL of eIF_3_d antibody (in pH 7.4 PBS) was applied to the modified GE surface and left to incubate for two hours. PBS (pH 7.4) was then used to wash the electrode surface in order to remove unbound protein moieties. After the immobilization phase, the surface was blocked with BSA (1.0 mg/mL) for 30 min to prevent nonspecific binding, a critical step for the sensor’s performance in real samples and in the presence of interfering molecules. The modified GE was then treated with eIF_3_d as the target analyte in varying concentrations (250, 200, 150, 50, 40, and 10 ng/mL in pH 7.4 PBS), and each sample was incubated for 30 min each to allow the biofunctional surfaces to selectively capture the analyte through the antibody/analyte recognition interaction. Each modification stage was characterized using electrochemical methods such as CV, DPV, and EIS in 5.0 mM Fe(CN)_6_^3–/4–^ containing 0.1 M KCl and pH 7.4 PBS, as well as surface analysis techniques such as SEM and XPS.

### Effects of Interfering Substances and Sample Application

A number of potential interferences, including AFP, HER2 as model protein biomarkers, and BSA due to its abundance in biological samples, particularly serum, were used to investigate selectivity, a crucial criterion in sensor performance. Instead of using the target biomarker (eIF_3_d), these chemicals were applied to GE modified as GE/antibody at concentrations of 40 ng/mL (in pH 7.4 PBS). Relative signal responses were computed by comparing the data with the eIF_3_d signal response (40 ng/mL).

eIF_3_d (40 and 150 ng/mL) was added to 1/100-diluted synthetic serum samples in order to examine the impact of the sample matrix and the suitability of the suggested system. Signal responses were then recorded when eIF_3_d-spiked solutions were applied to modified GE rather than eIF_3_d standards. The number of biomarkers in the samples was finally quantified by using a linear calibration curve.

## Results and Discussion

### Characterizations of the Synthesized MXene and F-Asp

Figure 1A shows the SEM image of the accordion-like structure of Ti_3_C_2_T_*x*_ MXene following the washing process. The image confirms the successful etching and exfoliation of Al from the parent MAX phase, resulting in loosely packed 2D nanosheets. Figure 1B presents the HR-TEM image of the Ti_3_C_2_T_*x*_ MXene, highlighting its well-defined layered structure at the nanoscale. The clear interlayer spacing observed in the image confirms the presence of surface functional groups (−F, −O, and −OH) introduced during chemical etching. The uniform alignment of the layers demonstrated their suitability for a wide range of applications.

The composition and surface properties of freshly prepared Ti_3_C_2_T_*x*_ MXene were analyzed through X-ray photoelectron spectroscopy (XPS). The XPS spectra are provided in Figure 1C, with peaks corresponding to Ti 2p, C 1s, O 1s, F 1s, and Cl 2p, indicative of the material’s composition and surface terminations. The Ti 2p spectra revealed peaks at 455 and 462 eV, which represent Ti–C and Ti–O bonding states. This was consistent with the expected structure of Ti_3_C_2_T_*x*_ MXene, where Ti–C bonds define the MXene’s carbide framework, while Ti–O bonds indicate the surface functional groups of MXene. The C 1s peak at 285 eV confirmed the presence of carbon in the Ti–C bond, along with possible contributions from adventitious carbon.

The presence of the O 1s peak at 528 eV further supports the existence of surface oxide (−O) and hydroxyl (−OH) groups, both of which are common surface terminations for MXenes synthesized through chemical etching. Additionally, the F 1s peak at 688 eV highlights the fluorine termination introduced during the etching process using a mixture of LiF and HCl. The Cl 2p peak, located at 200 eV, points to residual chlorine species from the etching process, suggesting that further washing could reduce the concentration of these byproducts. Finally, the observed Ti, C, O, F, and Cl peaks confirmed the successful fabrication of Ti_3_C_2_T_*x*_ MXene with characteristic surface terminations.

Synthesized F-Asp was characterized using a 1H NMR spectrum and IR spectroscopy (Figure 1D–F). The degree of aspartic acid substitution was quantified by 1H NMR spectroscopy through the addition of an internal standard and is summarized in Supporting Information.

### Surface Characterizations of the Modified Electrodes

**Figure 2:**
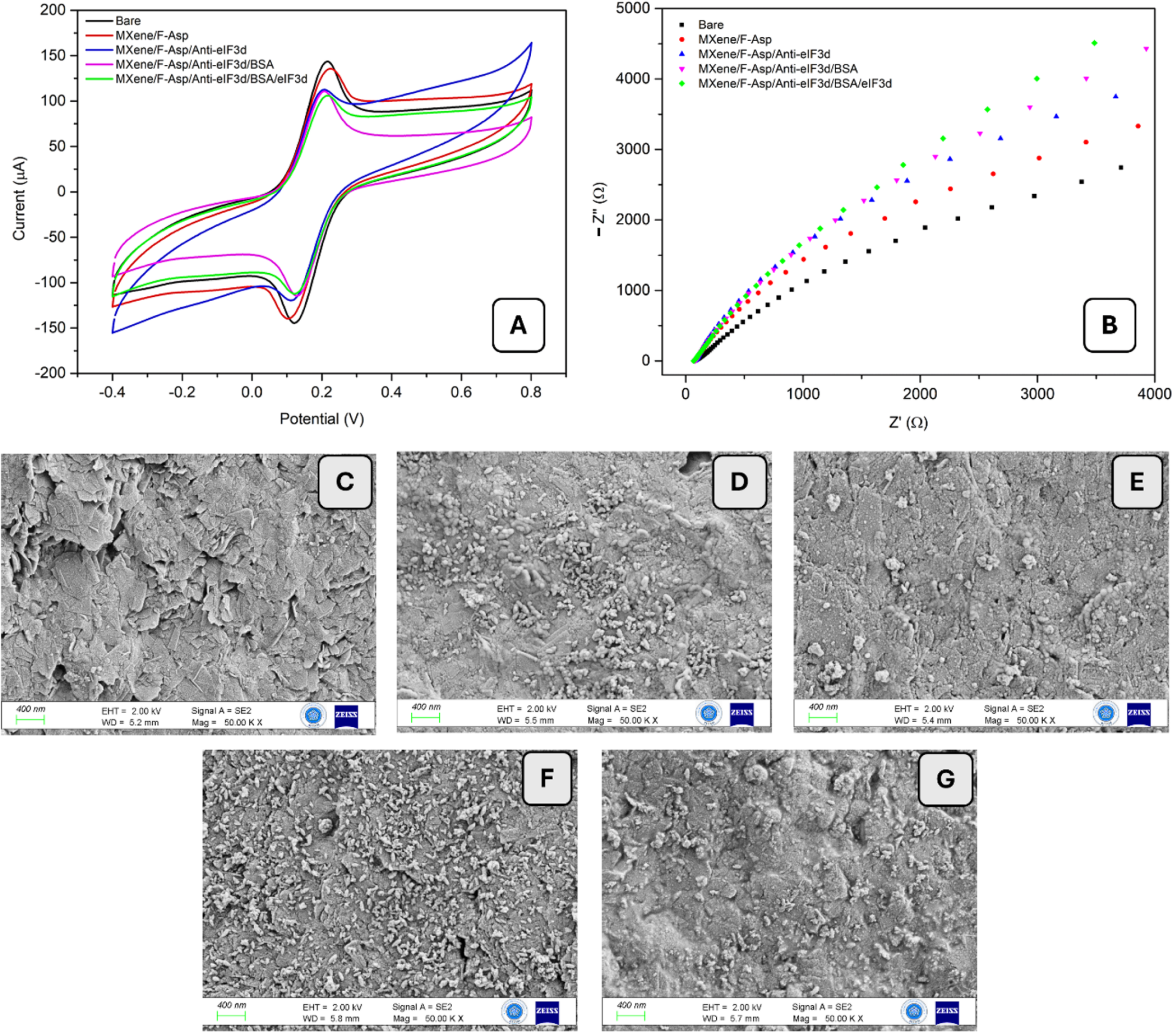
(A) CV and (B) EIS for bare GE; Mxene/F-Asp; Mxene/F-Asp/anti-eIF_3_d; Mxene/F-Asp/anti-eIF _3_d/BSA; and Mxene/F-Asp/anti-eIF _3_d/BSA/eIF _3_d (75 ng/mL) [measurements were carried out in the potential range from −0.4 to 0.8 V at the scan rate of 50 mV/s, 5.0 mM Fe(CN)_6_^3–/4–^]. SEM images of (C) bare GE; (D) Mxene; (E) F-Asp; (F) Mxene/F-Asp; and (G) Mxene/F-Asp/anti-eIF _3_d.

CV, EIS, XPS, and SEM analyses were used to confirm the surface alteration stages and the presence of analyte binding. MXene and F-Asp with carboxyl groups were initially added to bare GE. Then, the modified GE was treated with EDC/NHS, and the eIF_3_d antibody was applied to the surface. Prior to the addition of the analyte for the antibody/analyte binding reaction, the modified GE was blocked with BSA to prevent nonspecific interactions that could impair sensing performance in complex matrices such as tissue homogenates, serum, and urine. Aside from the analyte binding, each modification step resulted in larger structures as more molecules were integrated into the surface. These interactions block the redox probe from transferring to the electroactive area, resulting in a dip and difference in CV (Figure 2A) and DPV peaks, as well as an increase in EIS data. Figure 2B summarizes the findings of EIS measurements for surface characterization. The EIS findings were consistent with the CV results. The combination of MXene, F-Asp, and anti-eIF_3_d antibody on the electrode surface increased the resistance values (Rct). This expected finding indicated the presence of bulky structures, confirming successful attachment to the electrode surface.

Additionally, each surface modification has been verified through SEM analyses, as shown in Figure 2C–G. In contrast to the modified surfaces, the bare GE displayed a smooth surface shape (Figure 2C). Figures 2D–F demonstrate the homogeneous and well-distributed MXene and F-Asp clusters on GE with various dimensions. On the other hand, it was evident that eIF_3_d antibody molecules were effectively bound to the GE surface’s three-dimensional structure (Figure 2G). The primary advantage of this immobilization technique was that it preserved the antibody/antigen interaction, ensuring that the selective capture of the target analyte remained unaffected.

**Figure 3:**
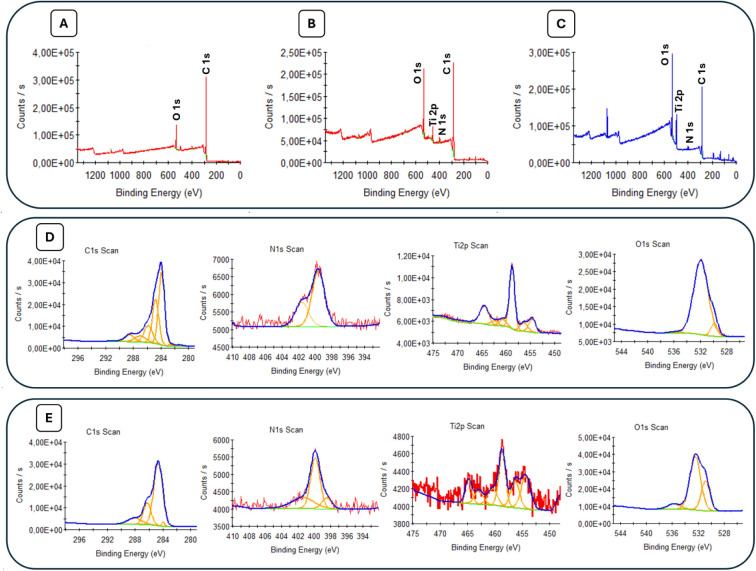
XPS survey spectra of the (A) bare GE, (B) GE-MXene-F-Asp, and (C) GE-MXene-F-Asp-anti-eIF_3_d with the labels C 1s, O 1s, N 1s, and Ti 2p. XPS high-resolution spectra of (D) GE-MXene-F-Asp and (E) GE-MXene-F-Asp-anti-eIF_3_d with the labels C 1s, N 1s, Ti 2p, and O 1s.

XPS analysis was very useful for understanding the structural nature and functional groups of the investigated compound. Analyzing the variations in elemental composition provided insights into the surface chemistry and functionalization of the materials. XPS analysis was used to monitor the functionalized surfaces, namely bare GE, GE-MXene-F-Asp, and GE-MXene-F-Asp-anti-eIF_3_d results of which are provided in Figure 3. The elemental compositions of the sample surfaces are tabulated and provided in Table 1. The presence of various elements in different proportions, along with changes in surface composition caused by specific treatments or modifications, was evident.

**Table 1:** Elemental Composition of the Bare-GE, GE-MXene-F-Asp, and GE-MXene-F-Asp-Anti-eIF_3_d Electrodes

% Element	Bare-GE	GE-MXene-F-Asp	GE-MXene-F-Asp-anti-eIF_3_d
C	84.49	69.55	55.63
N	1.73	2.33	1.97
Ti	ND	2.41	0.50
O	11.31	21.22	31.43
Si	1.33	1.47	1.26
P	0.49	0.79	4.63
Na	0.64	0.79	4.00
F	ND	1.45	0.59

Survey and high-resolution spectra of the elements are provided in Figure 3. The survey spectra of the samples revealed the existence of Ti, C, N, and O, except in the bare-GE-coded sample. The surface chemistry of the samples is understood through high-resolution spectra of C 1s, N 1s, Ti 2p, and O 1s. For the bare-GE-coded sample, the binding energy peaks of C 1s appeared at about 284.4 eV, 288.7 eV, 290.3 eV, and 293.2 eV, indicating the presence of C=C, C=O, and π–π∗ shakeup peaks, which correspond to the graphene structure.^32^ The C 1s high-resolution spectra of GE-MXene-F-Asp were fitted into eight components located at 281.4, 282.6, 284.0, 284.8, 285.8, 287.0, 288.5, and 290.8 eV, corresponding to Ti–C, Ti–C*, C=C, C–C, C–N, C–O, *O–C=O, C=O, and π–π∗ shakeup peaks.^33−39^ These peaks could indicate the presence of fullerene, MXene, and Asp functionalization on the surface of the bare-GE-coded sample. The GE-Mxene-F-Asp-anti-eIF_3_d-coded sample also has C 1s signals at 284.0 eV (C=C), 284.7 eV (C–C), 286.3 eV (C–N, C–O), 288.1 eV (C=O), and 291.91 eV (π–π∗ shakeup peak).^31−36,39−41^

MXene and F-Asp functional surfaces of bare-GE samples can be supported by Ti 2p and N 1s high-resolution spectra. The Ti 2p XPS peaks for the GE-MXene-F-Asp sample were observed at 454.6, 456.4, and 458.7 eV, while the peaks for the GE-MXene-F-Asp-anti-eIF_3_d sample appeared at 454.4, 456.3, and 458.7 eV. These signals were attributed to Ti–C, Ti–C*, and Ti–O bonding, respectively.^38^ Moreover, the N 1s signal at about 400 eV could be seen for all samples, but the peaks at about 401.6 and 398.5 eV correspond to the N–H bond in aspartic acid and *N=C–N–H structure in Anticor.^40,41^ The coatings on the surfaces of the samples were also supported by the O 1s signals. The peaks of the O 1s signals at 529.8, 530.5, and 532.3 eV were due to C–Ti–O, Ti–OH, and C–O.^37^

### Analytical Features of the MXene/F-Asp/Anti-eIF_3_d Biosensor

**Figure 4:**
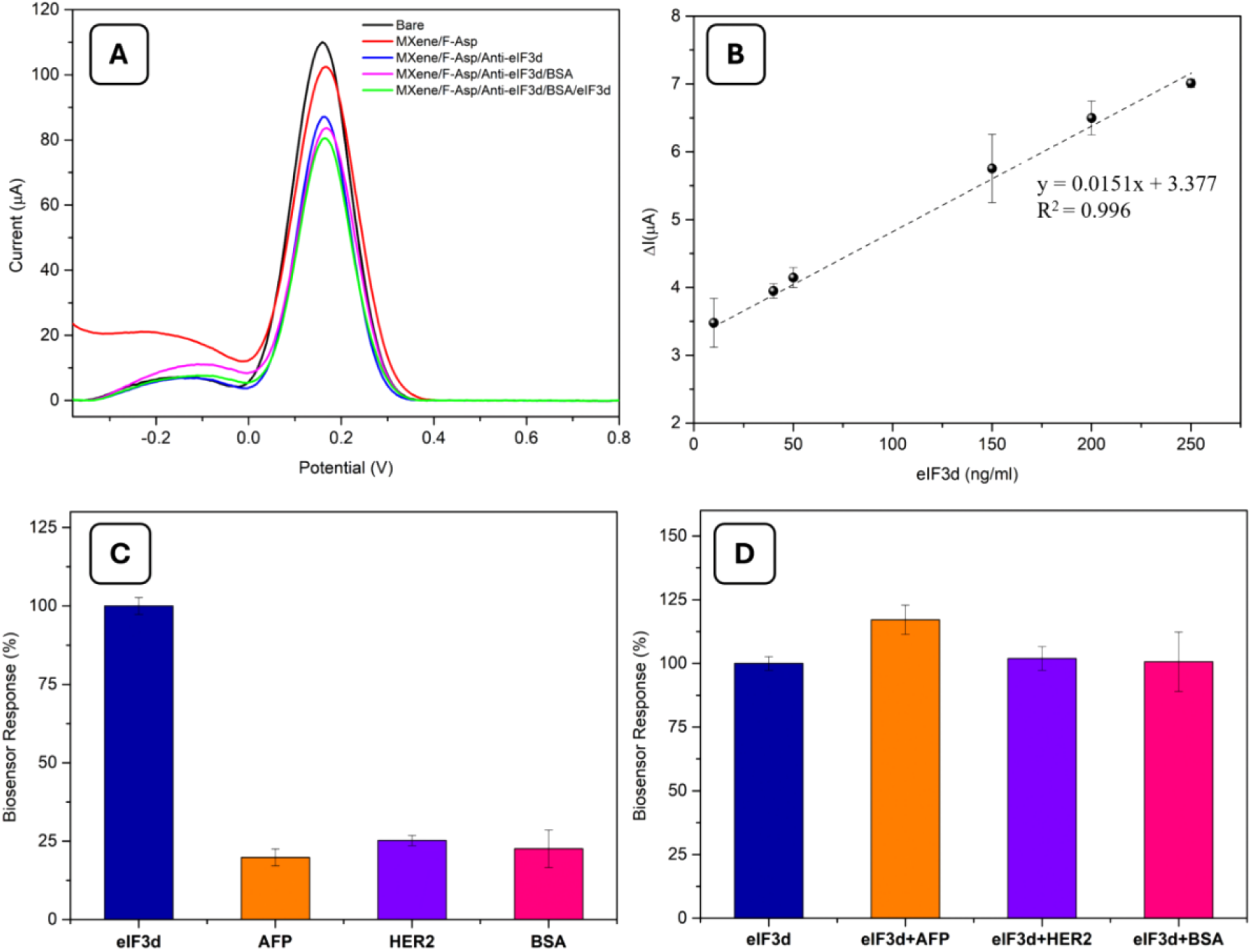
(A) DPV for bare GE; Mxene/F-Asp; Mxene/F-Asp/anti-eIF_3_d; Mxene/F-Asp/anti-eIF_3_d/BSA; and Mxene/F-Asp/anti-eIF_3_d/BSA/eIF_3_d (75 ng/mL) [measurements were carried out in potential range from −0.4 to 0.8 V at the scan rate of 50 mV/s, 5.0 mM Fe(CN)_6_^3–/4–^]. (B) Calibration curve for eIF_3_d biomarker [error bars were obtained by 3 measurements]. Biosensor response for (C) the various cancer biomarkers interfering molecules and (D) mixtures of various cancer biomarkers with eIF_3_ [all biomarker solutions and mixture solutions were prepared at 40 ng/mL concentrations].

Initially, DPV was used to observe the gradual alteration of GE (Figure 4A). The DPV results aligned with the CV and EIS results, as expected. Surface coverage by antibody binding and blocking with BSA resulted in a decrease in the current of DPV peaks. The concentration of the modifier and the biomolecules adsorbed on the electrode surface determines how well the immunosensor performs. To attain the best analytical performance in this instance, the quantity of MXene and F-Asp nanocomposite used as electrode layer components was investigated. Exceeding the modifier’s effective quantity may saturate the surface, causing the addition of the target molecule to produce an undetectable signal difference and obstructing the electron transfer channel. For varying amounts of the MXene/F-Asp nanocomposite, the DPV and peak current separation (ΔI) were measured. For subsequent studies, the optimal amount was determined to be 3.0 μL of 1/2 diluted MXene solution and 1 mg/mL of F-Asp.

A calibration graph was created between eIF_3_d (ng/mL) and current (ΔμA). Drops in DPV peak currents correlate with analyte concentrations, as can be seen in Figure 4B. The biosensor had a linear range of 10–250 ng/mL eIF_3_d. A departure from linearity was observed after 250 ng/mL. The analytical properties of the fabricated biosensor were determined. LOD values were calculated using the 3S/m criterion, based on the slope (m) of the calibration curves of the same biosensor. LOQ values were calculated using the 10S/m criterion, also based on the slope (m) of the calibration curves of the same biosensor. The LOD value was determined to be 0.14 ng/mL. Table 2 summarizes all of the obtained analytical properties for the modified GE.

**Table 2:** Some Analytical Characteristics of the Fabricated eIF_3_d Biomarker Biosensor

Linear Range	10–250 ng/mL
Slope	0.0151 μA/ng/mL
Intercept	3.377
Correlation coefficient	0.996
LOD	0.14 ng/mL
LOQ	0.42 ng/mL
Sensitivity	0.4771 μA·ng^–1^·mL·cm^–2^

The change in the biosensing system’s response signal is due to the selective capture of the target biomarker by the immobilized antibodies, which limits the transfer of the redox probe as a result of changes in surface characteristics caused by binding processes. Nonspecific binding of interfering substances with protein structures, such as other protein biomarkers, may potentially impact diffusion properties, resulting in a reduction in peak currents that are identical to those of the current target molecule (eIF_3_d). Interference tests were conducted by substituting the target protein biomarker with eIF_3_d and adding certain chemicals, such as AFP, HER2, and BSA, to investigate how this parameter affected the performance of the sensors. The eIF_3_d data were then compared with the obtained signal responses. For AFP, HER2, and BSA, the corresponding relative responses were less than 26% (19.82%, 25.22%, and 22.58%, respectively) (Figures 4C and Figure S1). Furthermore, Figure 4D shows that the relative response mixes of these substances with eIF_3_d were 117.11% for AFP + eIF_3_d, 101.96% for HER2 + eIF_3_d, and 100.64% for BSA + eIF_3_d. Therefore, it can be summarized that, in contrast to the other protein biomarkers, the eIF_3_d biomarker was selectively recognized by the fabricated biosensor.

### Sample Application of the MXene/F-Asp/Anti-eIF_3_d Biosensor

Two concentrations of eIF_3_d solutions (40 and 150 ng/mL) were added to the synthetic serum sample for sample application. The GE/MXene/F-Asp/Antibody/BSA surface was then directly treated with these solutions. Signals were compared to data from the normal eIF_3_d solutions (40 and 150 ng/mL) following the measurement of the signal under the previously indicated operating parameters (Figure S2). Table 3 summarizes the findings of the calculation of recovery and the relative standard deviation (RSD) values.

**Table 3:** Analysis Results for eIF_3_d in the Synthetic Serum Samples

Sample	Spiked with eIF_3_d (ng/mL)	Found eIF_3_d (ng/mL)	Recovery (%)	RSD (%)
Serum	40.0	40.99 ± 2.37	99.53	31.1
	150.0	149.29 ± 4.63	102.46	57.8

According to the literature, introducing nanoparticles into biosensor device design can enhance the analytical qualities of diagnostic tests (such as specificity, selectivity, or sensitivity).^42^ In particular, MXene, a 2D-layered material,^43−48^ and functional fullerenol^49,50^ have attracted significant interest in various research fields due to their wide range of potential applications. MXene-based sensors have demonstrated great promise in detecting cancer biomarkers, as their high conductivity and biocompatibility enable precise, real-time detection at ultralow concentrations. Highly active transducer surfaces that effectively integrate biomolecules and enable quicker access to analytes are necessary for the development of highly effective electrochemical biosensors. With its high density of functional groups, the ultrathin 2D nanosheet structure of few-layered MXene provides enhanced biomolecule loading and expedited analyte access.^44,45,47^

Similarly, fullerene and its derivatives have been used in biomedical applications since the functionalized fullerenol groups can suitably approach the target molecule covalently. Proper binding of fullerenol and biomolecules facilitates sufficient electron transfer between electrodes and the target analyte.^51^ Moreover, in our previous work, we demonstrated that amino acid-functionalized fullerenol derivatives were identified as novel sensor systems for acetylcholine detection and provided an excellent immobilization platform for AChE enzymes.^21^ Hence, herein, we present for the first time the combination of two different nanomaterials, MXene and F-Asp, for the development of an improved eIF_3_d biosensor. To the best of our knowledge, electrochemical biosensors based on MXene or fullerenol derivatives for eIF_3_d detection have not yet been reported. There is only one study in the literature, designed by Balaban et al.^52^ In their work, an electrochemical immunosensor platform was developed for eIF_3_d, and an LOD value of 50.43 ng/mL was estimated. Tables 4 and 5 compare the analytical performances of MXene- and fullerene-based electrochemical cancer biomarker sensors and techniques for eIF_3_d measurement, as reported in the literature. In summary, in our work, the combination of MXenes and F-Asp in the electrochemical biosensor technique provides excellent biosensing ability with its broad linear range, low LOD, high sensitivity, and applicability in serum samples for cancer biomarker eIF_3_d detection. This combination demonstrated superiority in terms of biosensory analytical properties compared to each material alone.

**Table 4:** Comparison of Analytical Performance of MXene and Fullerene Based-Electrochemical Cancer Biomarker Sensors in the Literature^a^

Modified Electrode	Analyte	Linear Range	LOD	Application	ref
ssDNA/AuHFGNs/PnBA-MXene/GCE	miRNA-122	0.01 aM–10 nM	0.0035 aM	Serum	(43)
MXene nanosheets/anti-CEA	CEA	1–25 pg/mL	3.2 pg/mL	Serum	(44)
ssDNA/AuNP@BLM/dMXene	BRCA1 gene	10 zM–1 μM	1 zM	-	(45)
SPCE/TiVC-MXene/Au NPs/Pb^2+^-aptamer	HER2	1.0–1200 pg/mL	50 fg/mL	Serum	(46)
Anti-CEA/*f-*Ti_3_C_2_-MXene/GCE	CEA	0.0001–2000 ng/mL	0.000018 ng/mL	Serum	(47)
Au–Ti_2_CT_*x*_ MXene/aptamer	Exosomes isolated from human lung carcinoma cells	1 × 10^2^ to 1 × 10^7^ particles/μL	58 particles/μL	Serum	(48)
GCE/fullerene C60-anti-HSP70	HSP70	0.8 pg–12.8 pg/mL	0.273 pg/mL	Serum	(49)
C60/MB/anti-PSA	PSA	15 pg/mL–8 ng/mL	1.7 pg/mL	Serum	(50)
Mxene/F-Asp/anti-eIF**_3_**d/BSA/eIF**_3_**d	eIF**_3_**d	**10–250ng/mL**	**0.14ng/mL**	**Serum**	**This work**

a AuHFGNs/PnBA: hierarchical flower-like gold, poly(*n*-butyl acrylate); CEA: carcinoembryonic antigen; AuNP@BLM: gold nanoparticle-decorated biomimetic bilayer lipid membrane; dMXene: delaminated Ti_3_C_2_T_*x*_; SPCE/TiVC-MXene/AuNPs/Pb^2+^-aptamer: screen-printed carbon electrode was modified with gold nanoparticle and TiVC MXene catalyst plus Pb^2+^ loaded aptamer; *f*-Ti_3_C_2_-MXene: aminosilane-functionalized Ti_3_C_2_-MXene; HSP70: heat shock protein 70; MB: Methylene Blue, PSA: prostate-specific antigen.

**Table 5:** Techniques for eIF_3_d Measurement in the Literature

Method	Sample Type	Linear Range	LOD	ref
Immunohistochemistry and Western blot	Tissue and cell culture	-	-	(53)
Immunohistochemistry	Tissue	-	-	(54)
Immunohistochemistry and Western blot	Tissue and cell culture	-	-	(55)
Mass spectrometry	Cell culture	-	-	(56)
ELISA	Blood, tissue, serum, etc.	0.25–8 ng/mL	0.1 ng/mL	(57)
ELISA	Plasma, serum	0.15–10 ng/mL	0.15 ng/mL	(58)
ELISA	Tissue homogenates, cell lysates, and other biological fluids	0.156–10 ng/mL	0.06 ng/mL	(59)

## Conclusions

We have effectively fabricated an electrochemical biosensor that uses anti-eIF_3_d to capture and quantify eIF_3_d. To achieve this, MXene and aspartic acid-functionalized fullerenol were utilized as supporting materials for cancer detection and the eIF_3_d biomarker. An immunosensor combining MXene and F-Asp with the eIF_3_d biomarker, rarely reported in the literature, has been developed. This combination provides a fast, user-friendly, and cost-effective platform suitable for cancer detection. It demonstrated a broad linear detection range (10–250 ng/mL) with a low limit of detection of 0.14 ng/mL, even in the presence of other proteins commonly found in serum samples. Thus, it offers a unique and cost-effective approach for detecting biomarkers and could serve as a model for various immunosensor studies, enabling single or multiple detection of different cancer types.

## Supporting Information Available

Characterizations of the synthesized F-Asp and DPV graphics of the Mxene/F-Asp/anti-eIF_3_d biosensor (PDF)

## Abbreviations

eIF_3_d: eukaryotic translation initiation factor 3 (eIF_3_) complex, subunit D
F-Asp: aspartic acid-functionalized fullerenol
GE: graphite electrode
LOD: limit of detection
ELISA: enzyme-linked immunosorbent assay
DPV: differential pulse voltammetry
CV: cyclic voltammetry
EIS: electrochemical impedance spectrometry
XPS: X-ray photoelectron spectroscopy
BSA: bovine serum albumin
