# Supplementary material for: Surface Engineering of MXene and Functional Fullerenols for Cancer Biomarker ‘eIF3d’
Source: Langmuir. 2025 Mar 21;41(12):8330–41. doi: 10.1021/acs.langmuir.5c00157 (PMC11966753; doi:10.1021/acs.langmuir.5c00157)
Supplement: Supplementary file 1 — la5c00157_si_001.pdf [file la5c00157_si_001.pdf]

Supporting Information

for

Surface Engineering of MXene and Functional Fullerenols for Cancer Biomarker 'EIF3D'

Dilek Soyler[a], Volkan Dolgun[b], Oyku Cetin[c], Yaqoob Khan[c], Emine Guler Celık[d], Salih Ozcubukcu[b], Husnu Emrah Unalan[c], Suna Timur[e,f]*, Saniye Soylemez[a]**

[a] Department of Biomedical Engineering, Faculty of Engineering, Necmettin Erbakan University, Konya 42090, Türkiye

[b] Department of Chemistry, Faculty of Science, Middle East Technical University, Ankara 06800, Türkiye

[c] Department of Metallurgical and Materials Engineering, Faculty of Engineering, Middle East Technical University, Ankara 06800, Türkiye

[d] Department of Bioengineering, Faculty of Engineering, Ege University, Bornova, Izmir 35100, Türkiye

[e] Department of Biochemistry, Faculty of Science, Ege University, Bornova Izmir 35100, Türkiye

[f] Central Research Testing and Analysis Laboratory Research and Application Center, Ege University, Bornova Izmir 35100, Türkiye

Corresponding-Authors,

*Suna Timur

Department of Biochemistry, Faculty of Science, Ege University, Bornova Izmir 35100, Türkiye

E-mail (suna.timur@ege.edu.tr)

**Saniye Soylemez

Necmettin Erbakan University, Department of Biomedical Engineering, 42090, Konya, Turkey

E-mail (saniye.soylemez@erbakan.edu.tr)

Table of Contents

1. Characterizations of the synthesized F-Asp

2. DPV graphics of the Mxene/F-Asp/Anti-eIF3d biosensor

1. Characterizations of the synthesized F-Asp

11.1 mg of F-Asp was dissolved in D_2O. To this solution, 10 μL (9.44 mg, 0.129 mmol) of DMF was introduced as internal standard. The solution was analyzed by ^1H NMR spectroscopy. As shown in Figure 1F, diastereotopic hydrogens of aspartic acid and aldehydic hydrogen of DMF were used as reference. The ratio of aspartic acid on F-Asp to DMF was calculated as 1/13.04, equivalent to 0.0767. This means 11.1 mg of F-Asp contains $0.129 \times 0.0767 = 0.00989\ mmol$ (1.32 mg) of aspartic acid.

Molecular weight of F-Asp can be written as

$$[720 + 133.1x + 17(26 - x)]\ g/mol \tag{S1}$$

Where;

- 720 is the molecular weight of C_{60} fullerene
- 133.1 is the molecular weight of aspartic acid
- x is the number of aspartic acid on F-Asp
- 17 is the molecular weight of OH
- 26 is the number of hydroxy groups on C_{60} fullerenol.
- In the synthesis, aspartic acids are replacing with OH groups. (26-x) refers that.

With this knowledge, the equation below can be written as

$$\frac{(133.1\ x) g/mol}{((720 + 133.1x + 17(26 - x)) g/mol} = \frac{1.32\ mg}{3.13\ mg} \tag{S2}$$

By solving the equation above, it was calculated that approximately 6 (x was calculated as 5.81.) aspartic acid was substituted to fullerenol.

Since initially, we started with 26 -OH group on the fullerenol and only 6 of them are functionalized by aspartic acid. It is concluded that all OH groups were not activated by 4-nitrochloroformate and -OH groups will still present on fullerenol along with aspartic acid on F-Asp.

2. DPV Graphics of the Mxene/F-Asp/Anti-eIF3d Biosensor

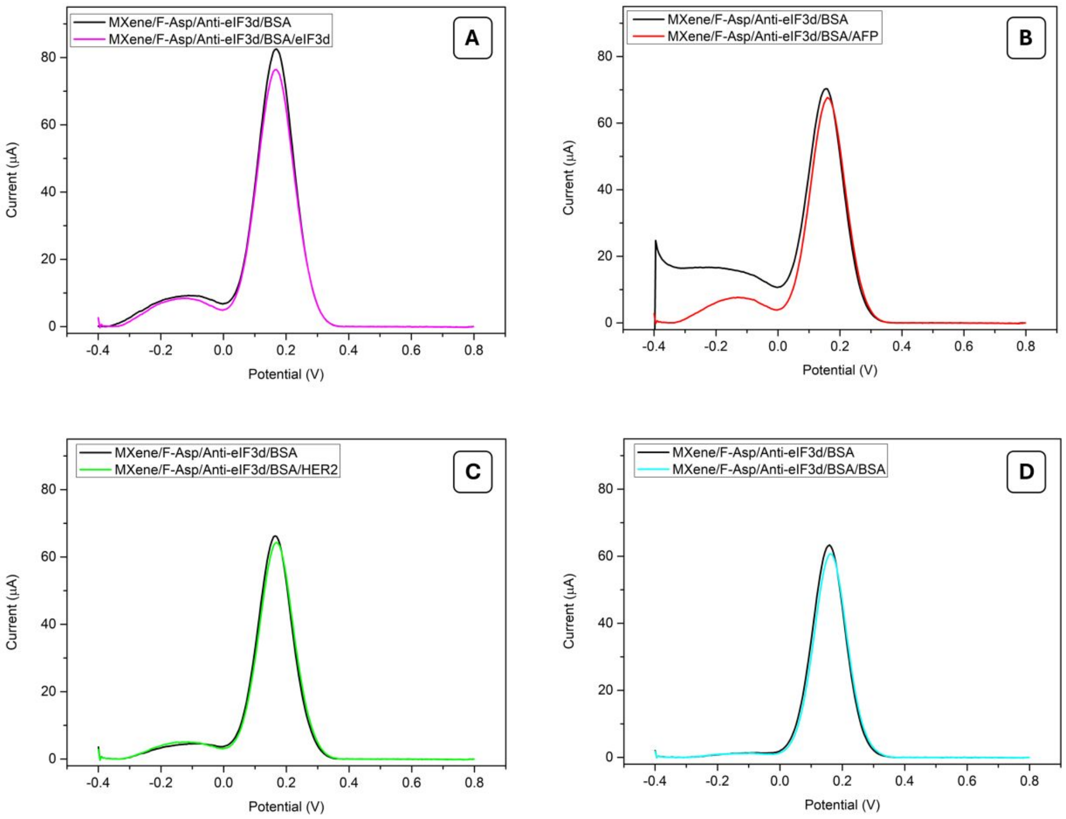

Figure S1. DPV curves obtained for 40 ng/ml (A) eIF3d, (B) AFP, (C) HER2, and (D) BSA used in interference study. All conditions were similar to Fig. 4C.

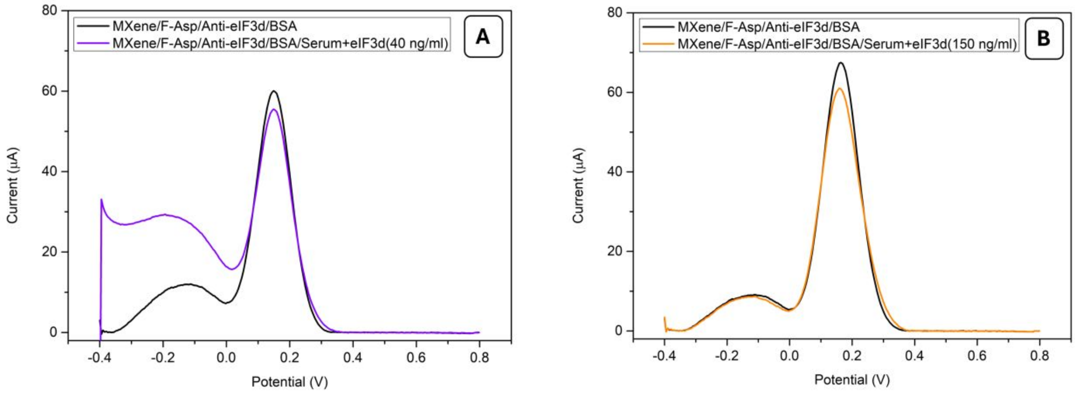

Figure S2. DPV curves obtained for (A) 40 ng/ml, and (B) 150 ng/ml eIF3d with serum used in sample application study. All conditions were similar to Table 3.
